# Supplementary material for: Horse Injury during Non-Commercial Transport: Findings from Researcher-Assisted Intercept Surveys at Southeastern Australian Equestrian Events
Source: Animals (Basel). 2016 Oct 25;6(11):65. doi: 10.3390/ani6110065 (PMC5126767; doi:10.3390/ani6110065)
Supplement: Supplementary file 1 [file animals-06-00065-s001.zip › S1_A Horse Transportation Survey_Float.pdf]

University of Adelaide Survey of Horse Injury During Transportation

ID

Event/location____ **Date**___/___/____ **Time**________

A) Float Driver Survey

Gender M ☐ F ☐ **Age last birthday** _______ y

1. **Were your horses towed or trucked to this event?** Y ☐ N ☐

2. **How often do you transport horses in a float for events and other activities?**

☐ Daily ☐ Monthly (how often per month?) ________

☐ Weekly (how often per week?) _________ ☐ Annually (how often per year?) __________

Months	Jan	Feb	Mar	Apr	May	Jun	Jul	Aug	Sep	Oct	Nov	Dec
	☐	☐	☐	☐	☐	☐	☐	☐	☐	☐	☐	☐

3. **What is the distance you travelled to transport horses to this event or activity?**

0 100 200 300 400 500 600 700 800 900 1000 >1000km

50 150 250 350 450 550 650 750 850 950

4. **What is the average km travelled when you transport horses for events and other activities?**

0 100 200 300 400 500 600 700 800 900 1000 >1000km

50 150 250 350 450 550 650 750 850 950

5. **What is the postcode of the place that the horses are kept?** ____ ____ ____ ____

6. **What is you level of training/licensure and experience as a driver (tick all that apply)?**

☐ Basic car licence ☐ Heavy vehicle license (medium rigid)

☐ Specific towing vehicle training ☐ Years driving _________ towing _________

7. **How many passengers do you typically carry when towing horses?**

0	1	2	3	4	5	6	7	8	Other
☐	☐	☐	☐	☐	☐	☐	☐	☐	____

8. **Are your passengers usually? (tick all that apply)**

Other riders	Your coach	Your partner	Children	Friends	Dogs	Other pets	Relatives
☐	☐	☐	☐	☐	☐	☐	☐

9. **On a scale of 1-7, how likely are you to answer a phone call whilst towing horses?**

	1	2	3	4	5	6	7	
Never	☐	☐	☐	☐	☐	☐	☐	Always

Dr Belinda Noble Prof Chris Riley 83131999

ID

10. On a scale of 1-7, how likely are you to <u>make</u> a phone call whilst towing horses?

	1	2	3	4	5	6	7	
Never	☐	☐	☐	☐	☐	☐	☐	Always

11. On a scale of 1-7, how likely are you to listen to music or the radio whilst towing horses?

	1	2	3	4	5	6	7	
Never	☐	☐	☐	☐	☐	☐	☐	Always

12. On a scale of 1-7, how likely are you to eat or drink whilst towing horses?

	1	2	3	4	5	6	7	
Never	☐	☐	☐	☐	☐	☐	☐	Always

13. How often do you typically take a rest break when towing horses?

Hourly	2 h	3 h	4 h	5 h	6 h	7 h	8 h	>8 h	Never
☐	☐	☐	☐	☐	☐	☐	☐	☐	☐

14. How much sleep have you had in the 24 hours prior to answering this survey?

None	1 h	2 h	3 h	4 h	5 h	6 h	7 h	8 h	>8
☐	☐	☐	☐	☐	☐	☐	☐	☐	☐

15. How much sleep have you had in the 48 hours prior to answering this survey?

None	1 h	2 h	3 h	4 h	5 h	6 h	7 h	8 h	9 h	10h	11h	12h	13h	14h	15h	16h	>16h
☐	☐	☐	☐	☐	☐	☐	☐	☐	☐	☐	☐	☐	☐	☐	☐	☐	☐

In order to assist us in preparing education materials on safe horse transportation, we would like to ensure that is prepared for the appropriate target audience. Some additional information about you would assist us.

16. What is your approximate income bracket?

<$25,000	$25,001 -$40,000	$40,001 -$60,000	$60,001-$80,000	$80,001 -$120,000	>$120,000
☐	☐	☐	☐	☐	☐

17. What is the highest level of education you have completed?

Year 10 or 11 high school	Year 12 high school	VET	TAFE	College	University degree	Graduate degree	Other
☐	☐	☐	☐	☐	☐	☐	☐

18. What is your most common source of information about horse health and care?

Friends	Coach or instructors	Magazines	Television	Email newsgroups	Internet websites	Facebook Twitter other social media	Veterinarian
☐	☐	☐	☐	☐	☐	☐	☐

ID

B) Horse travelling practice

1. How often do you perform a safety check on your float prior to travel?

0 Never	<25 %	25 – 50 %	51- 80 %	81 – 99%	100 %
☐	☐	☐	☐	☐	☐

2. Which of the following is typically included in your routine safety check (all that apply)

☐ None ☐Brakes ☐Lights ☐Tyre Pressure

☐Wheel bearings ☐Hydraulic fluid levels ☐Visual Integrity of the interior

☐ Other (list) ______________________________

3. What equine protective equipment do you typically use during transport (tick all that apply)

☐ None ☐Poll protection ☐Rugs ☐ Leg protection ☐ Tail wrap or bag ☐Other __________

4. How often do you rest (unload) your horse(s) during trips?

Hourly	2 h	3 h	4 h	5 h	6 h	7 h	8 h	>8h
☐	☐	☐	☐	☐	☐	☐	☐	☐

5. Are the horses' heads tied whilst travelling? Yes ☐ No☐

6. Do you provide feed to the horses when travelling?

No ☐ Hay only ☐ Concentrates only ☐ Both hay and concentrates ☐

7. What is the number of horses that fit in your usual horse truck?

1	2	3	4	5	6	7	8	9	10	Other
☐	☐	☐	☐	☐	☐	☐	☐	☐	☐	☐

8. What is the number of horses you generally tow?

1	2	3	4	5	6	7	8	9	10	Other
☐	☐	☐	☐	☐	☐	☐	☐	☐	☐	☐

9. Height (of largest horse transported)(please tick appropriate category)

☐<14hh ☐14.1– 15.3hh ☐16hh -17hh ☐>17hh

10. What is the estimated combined weight of horses usually towed?

☐<500kg ☐500-1000kg ☐1000 – 1500kg ☐1500-2500kg ☐2500-3500kg ☐>3500kg

11. Which breed(s) (up to 2) do you most commonly transport?

1.______________________ 2.______________________

12. What age are the horses that you regularly tow? (tick all that apply)

☐<2years ☐2-5years ☐5-10 years ☐11-15 years ☐ >15years

University of Adelaide Survey of Horse Injury During Transportation

ID

C) **Float details:** (Driver or surveyor to complete)

1. **What is the brand and year of manufacture of your float?**

   Brand______________________________ Year of manufacture___________________________

2. **What is the float type?**

Straight load ☐ Angle load ☐ Gooseneck ☐ Other (specify) ☐ ______________________

3. **What is the maximum number of horses that the float is capable of carrying?**

1	2	3	4	5	6	7	8	9	10	Other
☐	☐	☐	☐	☐	☐	☐	☐	☐	☐	☐

4. **What type of braking system is fitted to the float?**

   ☐ Hydraulic ☐Electric ☐Both ☐None ☐Other ______________________

5. **What type of suspension is fitted?**

   ☐ Leaf spring ☐ Gas struts ☐ Coil spring☐ Rocker/roller ☐ Other __________________

6. **How many axles are present?** ☐ 1 ☐ 2 ☐ 3

7. **What is the trailer hitch tow weight rating?**

   ☐<1500kg ☐1500-3000kg ☐3000-6000kg ☐>6000kg ☐Unknown

8. **Tailgate configuration?** ☐Single (half) gate ☐Split gate ☐Fully enclosed

9. **Is a chest bar present**

   ☐No ☐ Yes: ☐ Fixed ☐ Movable ☐ Quick release

10. **Breeching closure**. ☐Chains ☐Small bars ☐Half length doors ☐Full length doors

11. **Are bay dividers installed?** ☐ No ☐Partial ☐ Full length (solid) ☐Full length (rubber)

12. **Are living spaces/ tack box/rug racks present? (tick all that apply)**

    ☐ None ☐ Moveable Tack Box ☐ Rug Rack ☐ Living space ☐ Front storage space

13. **Ventilation** ☐ None ☐ Passive (ie pop-up vents/ windows etc) ☐ Active (ie powered fan)

ID

D) Towing Vehicle Details

1. **What vehicle did you tow with today or what is your primary towing vehicle for horse transport if you did not transport horses today?**

Make ______________________ Model ____________________ Year manufactured _________

Cylinders 4☐ 6 ☐ 8 ☐ Engine size ____________ L Manual ☐ Automatic ☐

2. **Did you perform a safety check of your vehicle prior to this trip?**

☐ Yes ☐No ☐ N/A (if did not tow today)

3. **Where is the hitching point for towing your trailer on this vehicle?**

☐Rear ☐Mid back axle (ie gooseneck) ☐ N/A (truck)

4. **Does your towing vehicle have torsion bars?**

☐Yes ☐No

5. **Towbar rating (if known)**

____

6. **Do you perform routine checks of the vehicle prior to travel with horses?**

0 Never	<25 %	25 – 50 %	51- 80 %	81 – 99%	100 %
☐	☐	☐	☐	☐	☐

7. **Which of the following is included in your routine check (all that apply)**

☐ None ☐Brakes ☐ Tyre Pressure ☐ Visual Integrity of the interior

☐ Lights ☐Wheel bearings ☐Hydraulic fluid levels ☐ Other (list) ________________

8. **When was the vehicle last maintained/serviced by a licensed mechanic?**

☐<3 months ☐3-6months ☐6 -12 months ☐>12months ☐ Never
