# Supplementary material for: Horse Injury during Non-Commercial Transport: Findings from Researcher-Assisted Intercept Surveys at Southeastern Australian Equestrian Events
Source: Animals (Basel). 2016 Oct 25;6(11):65. doi: 10.3390/ani6110065 (PMC5126767; doi:10.3390/ani6110065)
Supplement: Supplementary file 1 [file animals-06-00065-s001.zip › S3_Horse transport accident survey.pdf]

University of Adelaide Survey of Horse Injury During Transportation

Event/location____ **Date**___/___/____ **Time**________ ID

1. **Has a horse experienced a major or minor injury whilst being transported in your horse float or truck, including injury to a horse during an otherwise uneventful trip?**

   No ☐ Yes, being transported in: a float ☐ or truck ☐

2. **How many years ago did this injury occur?**

   ≤1 ☐ >1 to 2 ☐ >2 to 3 ☐ >3 to 4 ☐ >4 to 5 ☐ >5 to 6 ☐ >6 to 7 ☐ >7 to 8 ☐ >8 to 9 ☐ >9 to 10 ☐ >10 to 15 ☐ >15 to 20 ☐

3. **During what stage of the journey did this injury occur?**

   Loading ☐ Loaded (stationary) ☐ Loaded (moving) ☐ Unloading ☐

4. **At approximately what speed was your vehicle travelling?(mark)**

   0 10 20 30 40 50 60 70 80 90 100 110 120 >120 kph

5. **What was the time of day the accident occurred? (mark)**

   12.01am 2am 4am 6am 8am 10am 12noon 2pm 4pm 6pm 8pm 10pm 12pm

   1am 3am 5am 7am 9am 11am 1pm 3pm 5pm 7pm 9pm 11pm

6. **What were the weather conditions (tick all that apply)**

   Fine ☐ Cloudy ☐ Overcast ☐ Fog ☐ Raining ☐ High winds ☐ Hailing ☐ Wet road ☐ Very hot ☐

7. **How many passengers were in the car at the time of the accident?**

   0 ☐ 1 ☐ 2 ☐ 3 ☐ 4 ☐ 5 ☐ 6 ☐ 7 ☐ 8 ☐ Other ____

8. **What was the distance travelled on that trip prior to the accident? (mark)**

   0 100 200 300 400 500 600 700 800 900 1000 >1000km

   50 150 250 350 450 550 650 750 850 950

9. **What was the duration of that trip? (mark)**

   0 1 2 3 4 5 6 7 8 9 10 11 12 >12h

10. **How long had you been driving continuously at the time of the accident since the last rest break?**

    0 1 2 3 4 5 6 7 8 9 10 11 12 >12h

11. **What were the make, model and year of manufacture of the tow vehicle or truck being used?**

    Make ____________________ Model ____________________ Year manufactured __________

    Cylinders 4☐ 6 ☐ 8 ☐ Engine size ____________ L Manual ☐ Automatic ☐

Dr Belinda Noble Prof Chris Riley 83131999

University of Adelaide Survey of Horse Injury During Transportation

ID ☐

For trucks

Tray/single body ☐	Gross vehicle weight or mass __________ kg	Number of axles ___________
Semi-trailer ☐	Gross combined weight rating__________ kg	Air brakes? Yes ☐ No ☐

12. If a float was towed, what type was it?

Straight load ☐ Angle load ☐ Gooseneck ☐ Other (specify) ☐________________________

13. What was the maximum number of horses that the float or truck was capable of carrying?

1	2	3	4	5	6	7	8	9	10	Other
☐	☐	☐	☐	☐	☐	☐	☐	☐	☐	☐

14. How many horses were carried during the trip during which the accident occurred?

1	2	3	4	5	6	7	8	9	10	Other
☐	☐	☐	☐	☐	☐	☐	☐	☐	☐	☐

15. If the vehicle or float was damaged in the accident, please indicate where below (tick all that apply)

		Front	Side(s)	Rear	Roof	Chassis	Write-off	Other
Float	☐	☐	☐	☐	☐	☐	☐	_________________________
Vehicle	☐	☐	☐	☐	☐	☐	☐	_________________________

16. Please mark the numbers corresponding to the regions of the horse injured on the schematic below

No injury	1	2	3	4	5	6	7
☐	☐	☐	☐	☐	☐	☐	☐

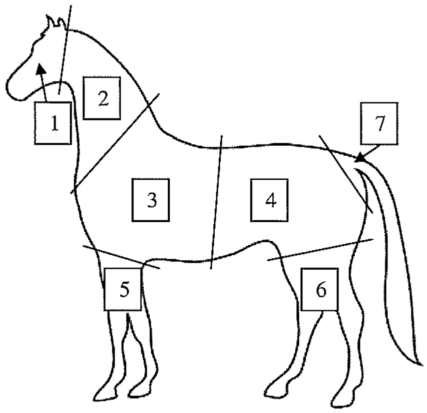

17. Please rank the injury to the horse(s)

☐ None
☐ Minor (requiring owner administered first aid)
☐ Moderate (requiring veterinary attention)
☐ Marked (requiring intensive veterinary management)
☐ Severe (causing death/ requiring euthanasia of horse)

18. Please indicate if the horse(s), float or vehicle were insured at the time of the accident and if a claim was made as a result?(tick all applicable)

Float		Vehicle		Horse(s)	
insured? ☐	claim made? ☐	insured? ☐	claim made? ☐	insured? ☐	claim made? ☐

19. Please provide a brief outline of the particulars of accident

____
____
____
____
____
____

Dr Belinda Noble Prof Chris Riley 83131999

ID

20. Which factors do you believe may have contributed to the accident?

**Collision with**	**Driver**		**Mechanical failure**
☐ another vehicle	☐fatigue		☐towing vehicle
☐a stationary object	☐illness		☐float
☐wild or native animal	☐error		☐truck
☐domesticated animal	☐inexperience		
**Horse**	distraction by	☐passengers	☐**Weather conditions**
☐loss of footing		☐telephone	☐**Road conditions**
☐injury by other horse		☐music	☐**Time of day**
☐poor behaviour		☐other	
☐injured by part of transport vehicle		☐horses being transported	

Other factors

____

____

____

____

____

____
